# Impact of the COVID-19 pandemic on long-term trends in youth depression and anxiety

**DOI:** 10.1007/s44192-025-00311-5

**Published:** 2025-12-30

**Authors:** Mark W. G. Bosmans, Marjonneke de Vetten-Mc Mahon, Julie A. C. Penders, Imme J. Rahmon, Elske Marra, Bob Inja, Tanja van der Marel, Michel L. A. Dückers

**Affiliations:** 1 Netherlands Institute for Health Services Research (Nivel), Utrecht, The Netherlands; 2 National Institute for Public Health and the Environment (RIVM), Bilthoven, The Netherlands; 3 Faculty of Behavioural and Social Sciences, University of Groningen, 9700 AB Groningen, The Netherlands; 4 ARQ National Psychotrauma Centre, Diemen, The Netherlands

**Keywords:** Youth mental health, Anxiety, Depression, Long term trend, COVID-19, Systematic narrative review

## Abstract

Adolescent depression and anxiety have been rising in recent decades. Although reviews point at deterioration of youth mental health during the COVID-19 pandemic, how this relates to pre-pandemic trends is unclear. This narrative review examines whether the COVID-19 pandemic has impacted the long-term development of depression and anxiety among adolescents. A systematic search in PubMed/Medline, PsycINFO, and Embase identified longitudinal or repeated cross-sectional studies using random sampling among youth (11–25 years). Out of 4,761 articles, nine met inclusion criteria. Most studies (7 out of 9) reported rising anxiety and/or depression rates pre-pandemic. During the pandemic, two trends emerged: five studies observed an amplification of pre-existing increases, while four reported a continuation of prior trends, none pointing at improvement. The majority of studies support the hypothesis that COVID-19 exacerbated existing increases in anxiety and depression among adolescents and young adults. Additional longitudinal studies with randomized, general population samples are needed to determine post-pandemic developments.

**Supplementary Information:**

The online version contains supplementary material available at 10.1007/s44192-025-00311-5.

## Introduction

Adolescence is a particularly vulnerable time for the development of mental health disorders, especially depression and anxiety [1]. These disorders can significantly affect overall health and functioning across various areas of life well into adulthood [2]. Approximately one in seven individuals aged 10–19 experiences a mental disorder, contributing to 13% of the global disease burden in this age group [3]. In recent decades, depression has been on the rise among adolescents and young adults, particularly in developed countries. Between 1990 and 2019, Disability-Adjusted Life Year (DALY) rates for depressive disorders increased significantly among youth (+ 23%) in high Socio-Demographic Index (SDI) countries, compared to a decline in countries with lower SDI levels (−1,7% to −4,2%) [4, 5]. During the same period, the incidence and global burden of anxiety disorders among adolescents also steadily increased in developed countries, while decreasing trends were observed in less developed countries [6]. Although no definitive explanation exists for this discrepancy, several contributing factors have been suggested. In developed countries, greater awareness of mental health may reduce stigma and improve mental health literacy, enabling individuals to more readily associate distress or atypical behavior with mental health conditions and seek a diagnose or self-diagnose. These countries also typically have better access to diagnostic tools and mental healthcare services. Additionally, country-specific cultural and social factors—such as an emphasis on individual (academic) achievement, parenting styles, and the pervasive influence of social media—may play a role [4, 6–8].

Previous studies have shown that disasters and crises can affect the mental health of those exposed. Common psychological problems are posttraumatic stress, depression and anxiety. Although the majority typically cope well in the aftermath of a disaster, a significant minority experiences psychological distress [9–12]. A smaller minority develops lasting mental health issues [11, 13, 14]. A consistent finding in post-disaster studies is that youth (adolescents and young adults) are more likely than other age groups to be severely psychologically affected [10–12]. Notably, a systematic review by Newnham et al. [11] examining the prevalence of PTSD, depression and anxiety following disasters and pandemics found that while PTSD levels tend to decrease over time, depression and anxiety rates remain elevated for years post-exposure, including among children and adolescents .

The recent Coronavirus disease (COVID-19) pandemic affected the total population either directly through the SARS-CoV-2 infection and the associated threat, or indirectly through the measures taken to counter the spread of the disease. These effects included hospitalizations, fatalities, long-term health problems, delayed care, decreased physical activity, and social isolation [15–20]. Meta-analyses and reviews indicate that youth experienced a deterioration of mental health during the pandemic specifically among youth [21–24], particularly with increased levels of depression and/or anxiety [25–30].

The COVID-19 pandemic shares attributes with other disasters and crises in that it threatened health and life and disrupted daily routines and critical infrastructure. At the same time, it was unique in several ways. First, it affected not just localized populations, but people worldwide. Second, in addition to the threat of illness of death, pandemic-related countermeasures—designed to limit the spread of the virus—severely restricted normal social interactions and disrupted factors related to mental health, such as daily routines and family finances [31]. For adolescents, these restrictions meant prolonged school closures, reduced contact with peers (e.g., at sports clubs), and the loss or virtualization of key developmental milestones such as graduation ceremonies and university introductions. Healthy adolescent development relies on exploring social environments beyond the family and forming more mature relationships [32, 33]. Both processes were significantly disrupted by pandemic-related restrictions, leaving adolescents particularly vulnerable.

The well-established impact of disasters and crises on adolescents and young adult mental health, combined with this group’s heightened sensitivity to disrupted social interactions, suggests that the pre-existing upward trend in depression and anxiety among adolescents may have accelerated during the COVID-19 pandemic. However, research on the pandemic’s impact on adolescent mental health has several limitations, making it difficult to assess long-term effects. Most studies used cross-sectional designs, began data collection only after the pandemic began, did not use representative population samples, or focused on different populations or research questions [28, 30]. Building on these findings, this study aims to investigate how the COVID-19 pandemic has influenced the long-term prevalence of depression and anxiety among adolescents and young adults globally.

## Methods

### Guidelines

The current study followed the Preferred Reporting Items for Systematic Reviews and Meta-Analyses (PRISMA) guidelines. Ethics approval was not required for this systematic review, as the data were retrieved and analysed from previously published studies.

### Literature search

The following scientific databases were searched for articles: PubMed (including all Medline records), PsycINFO and Embase. Keywords were based on variations of (1) prevalence, incidence, or epidemiology, (2) multiple measurements or longitudinal design, (3) youth or young adults, and (4) depression and/or anxiety symptoms. See the supplementary table for the search strings used per database. Relevant reviews and meta-analyses identified during screening, were also examined for possible relevant articles, by using the backwards snowballing method.

### Inclusion and exclusion criteria

Articles were included if studies (1) were published in or accepted by scientific journals between January 1, 2020 and January 31, 2024, (2) applied an observational epidemiological research design, (3) had a longitudinal or repeated cross-sectional design, (4) used a random sample, (5) focused on youth aged 11–25 or included a subgroup within this range, (6) reported depressive and/or anxiety symptoms as continuous (mean) scores or binary outcomes with a specified cut-off point, (7) included at least two measurements before the COVID-19 pandemic and one during the pandemic, and (8) had a study duration of at least two years. No language criteria were applied to the search. Exclusion criteria were; (1) studies reporting diagnosed conditions in clinical or patient samples, (2) studies focused on pathogenesis and virological mechanisms of COVID-19, (3) animal studies, and (4) review articles, editorials and gray literature (i.e., reports or dissertations).

### Missing data

Missing data were requested from the authors of eight articles. Except for three [34–36], the studies were excluded due to crucial missing data (e.g., mean scores, sample size, timing of measurement).

### Screening procedure

Screening occurred in two phases. First, titles and abstracts were reviewed by three screeners (JP, BI, and TM). In the second phase, full texts were screened. All articles were screened independently at least twice by two different researchers during both phases. Disagreements were resolved through discussion; if necessary, a third researcher (MB/MV) was consulted. When in doubt, articles were retained for full-text screening. For studies using the same dataset, the version reporting both continuous and binary outcomes was included. If not applicable, the study with a larger sample size/or more measurement points was selected. For these reasons, four studies were excluded [37–40].

### Quality assessment

An 8-item tool adapted from NIH Quality Assessment Tool for Observational Cohort and Cross-Sectional Studies was used for quality assessment. Assessed topics included sample characteristics (representativeness and size), response rates, missing data, validity of instruments for measuring anxiety and depressive symptoms, consideration of confounding variables, and reporting of demographic information. See Table 1 for details.

**Table 1:** Overview study characteristics and main findings

First author/year	Country	Design	Quality criteria meta^a^	Outcome	Measure	Reporting	No. of pre-COVID measurements	No. of COVID measurements	Timing of COVID measurement	Sample size COVID measurement	Pandemic circumstances during measurement	Pre-pandemic trend	Trend during pandemic*
Alzueta et al. [34]	USA	Longitudinal	1,2,6,8,9 (5)	Depression	Self-reported; CESD-R-10	Mean	7	1	2020, months unknown	525	Stay at home rules, school closures and social distancing measures in effect in some regions part of the time. Exact circumstances during data collection unclear	Increasing	Amplification of increase
Gohari et al. [44]	Canada	Longitudinal	1,2,5,6,8,9 (6)	Depression + Anxiety	Self-reported; CESD-R-10 + GAD-7	Mean + prevalence significant depressive symptoms and generalized anxiety (GA) (usual cut-off ≥ 10)	2	1	2020–2021, months unknown	3.447	Lockdown, yet schools were open most of the time, with face-masks mandatory. Most of this period restaurants and bars closed, only outdoor recreation allowed. Public gatherings prohibited. In some regions only contacts within social bubble allowed and non-essential travel prohibited	Increasing	Amplification of increase
Kiviruusu et al [45]	Finland	Repeated cross-sectional	1,2,4–6,8,9 (7)	Anxiety	Self-reported; GAD-7	Prevalence generalized anxiety (GA) (usual cut-off ≥ 10)	4	1	2021 (Mar-May)	158.436	Most adolescents from high schools and vocational schools had spent almost the entire 2020–2021 school year in online instruction but recently returned to in-person learning, whereas younger students in comprehensive schools had spent virtually the whole 2020–2021 school year in on-site instruction	Increasing	Amplification of increase
Goodwin et al [48]	USA	Repeated cross-sectional	1,2,4,8 (4)	Depression	Self-reported; Adapted from National Comorbidity Survey-replication	Prevalence lifetime and past-year major depressive episode (MDE) (usual cut-off ≥ 5 of 9 symptoms for MDE including either depressed mood or loss of interest or pleasure in daily activities	2	1	2020, months unknown	24.983	Full lockdown: stay at home unless absolutely necessary. Schools were closed, public gatherings prohobited, work-from-home-advice intensified: only go to work when this is absolutely necessary	Increasing	Continuation of increase
Lipson et al [35]	USA	Repeated cross-sectional	1,5,6,8,9 (5)	Depression + Anxiety	Self-reported; PHQ-9 + GAD-7	Prevalence symptoms of generalized anxiety (GA) and depression (usual cut-off ≥ 10)	6	1 (1 measurement both before and during COVID omitted from analyses)	2020–2021, months unknown	359,777 over all 8 measurements	Stay at home rules, school closures and social distancing measures in effect in some regions part of the time. Exact circumstances during data collection unclear	Increasing	Continuation of increase
Thorisdottir et al [47]	Iceland	Longitudinal	1,2,4–6,8,9 (7)	Depression	Self-reported; Depression dimension of SCL-90	Mean	2	1	2020 (Oct-Nov)	6.136	Strict physical-distancing rules. Secondary schools (students 16–18 years) limited to online teaching. Students ≤ 16 s) continued to receive on-site learning in school	Increasing	Amplification of increase
Trompeter et al [36]	Australia	Repeated cross-sectional	1,5,6,8,9 (5)	Depression	Self-reported; CES-D	Mean	5	1	2020 (May-Aug)	159	Data were collected in New South Wales, where measures were eased significantly at the time: schools had been reopened and most other restrictive measures were lifted	Fluctuating	Continuation of fluctuation (decrease)
Von Soest [46]	Norway	Repeated cross-sectional	1,2,4–6,8,9 (7)	Depression	Self-reported; Depressive Mood Inventory	Mean	7	1	2021 (Jan-Mar)	86.597	Some school closures, but not of all schools at once. Schools that were closed did not participate in the study. Norway had a relatively low rate of COVID-19-related deaths and lower infection rates	Increasing	Amplification of increase
Wang et al [43]	China	Longitudinal	1,2,4–6,8,9 (7)	Depression + Anxiety	Self-reported; CES-D + GAD-7	Mean + Prevalence anxiety and depressive symptoms (usual cut-off CES-D score ≥ 16; GAD-7 ≥ 5 considered indicative of depression/anxiety symptoms)	2	1	2020 (Oct-Dec)	1.790	Schools had been reopened 5–7 months before data-collection	Stable	Continuation of stable trends

^a^ “Quality criteria met” is based on an 8-item quality assessment tool adapted from “The National Institute of Health (NIH) Quality Assessment Tool for Observational Cohort and Cross-Sectional Studies”. *Defined sample*: (1) Has the study defined eligibility and exclusion criteria for their sample; and time period (dates) and location (s) of recruitment and assessment?; *Representative sample*: (2) Is the sample representative of a defined population? (i.e., was everyone included who should be, and is this sample generalizable for the population of interest); *Adequate sample*: (3) Was a sample size justification or power description provided?; *Participation*: 4.Was the participation/response rate of eligible persons at least 50%?; *Missing data*: 5. Does the study mention missing data and account for how they were treated in the analysis?; *Valid instruments*: 6. Did the study use validated instruments for the assessment of main outcomes (dependent variables)?; *Subjective vs. Objective measures*: 7. Did the studies only use self-reported questionnaires (0), objective measures, for example clinical diagnoses, medical records (1) or both for the same outcome (2); *Confounding variables*: 8. Were confounding variables taken into account in the analysis?; *Demographic information*: 9. Does the study report descriptive/demographic data of the sample? (such as age, gender or education); *Loss to follow-up (only for cohort/longitudinal studies)*: 10. Was loss to follow-up after baseline 20% or less? (Source: National Institute of Health Quality Assessment Tool for Observational Cohort and Cross-Sectional Studies)

### Data extraction

From the included studies, the following information was extracted: the mental health outcomes studied (mean scores of depressive or anxiety symptoms and prevalence of youth exceeding cut-off points indicating significant symptoms or probable disorder), study design (longitudinal or repeated cross-sectional), population type, number and dates of pre- and post-COVID measurements, study region, instruments used, and sample sizes. For studies reporting continuous outcomes, sample size, mean scores, and standard deviations were extracted. For studies reporting prevalence, both the prevalence and the cut-off values used were collected. Data extraction was performed by three researchers (JP, BI, and TM) and double checked by at least one of the other researchers (MB/MV).

### Narrative synthesis

Due to heterogeneity among the included studies, a narrative synthesis was conducted instead of a meta-analysis. This allowed for identifying trends in depression and anxiety before and during the pandemic among adolescents and young adults, while also considering contextual factors that may explain observed outcomes. For visual comparisons of mean symptom levels across studies, the means from nine measurement series from six studies (reporting depression, anxiety or psychological distress at different time points) were transformed using the Percentage of Maximum Possible (POMP) scores (see Supplement Data File) [41]. The transformation enabled comparison across instruments with different scoring scales.

## Results

**Fig. 1:**
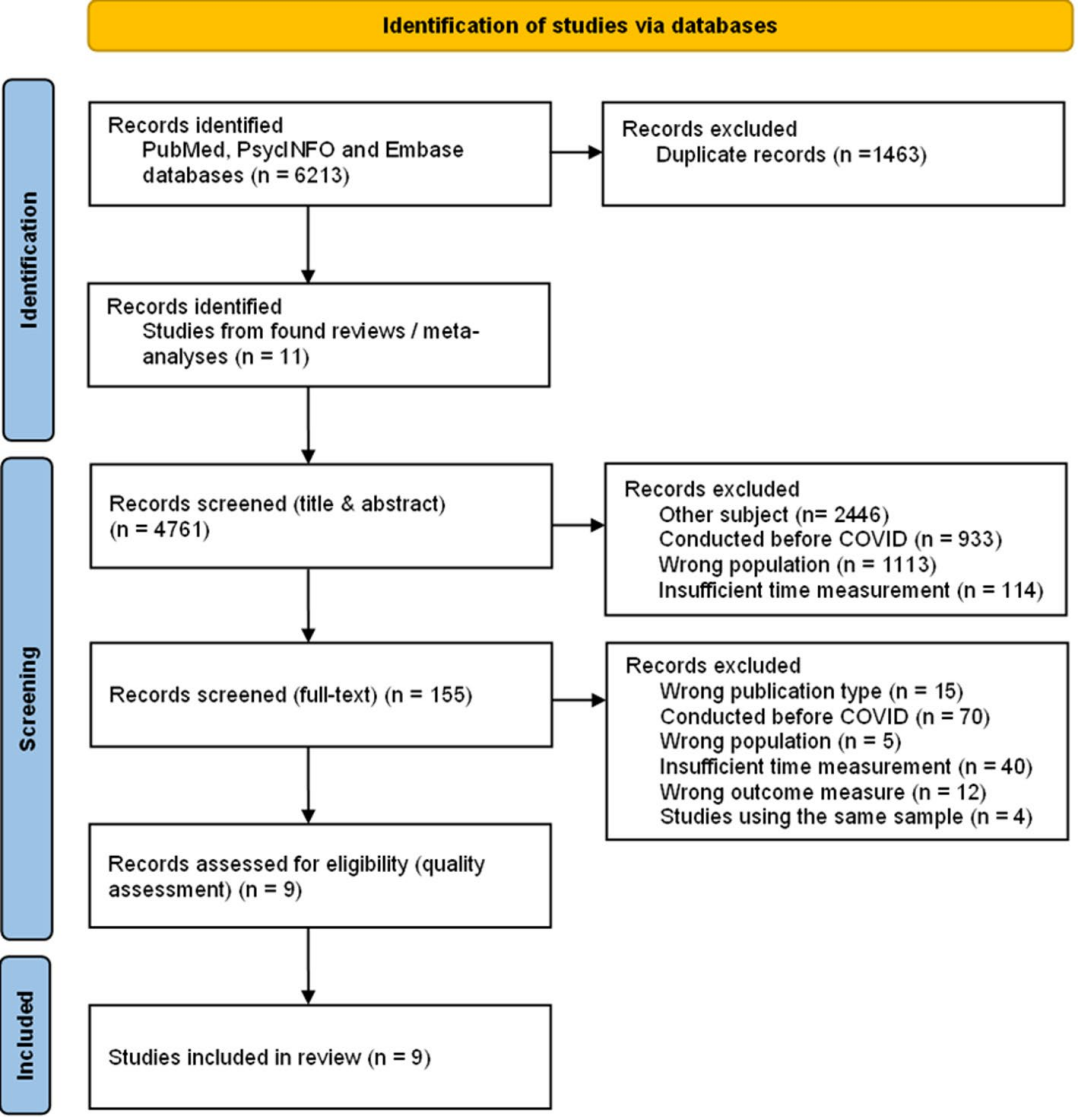
PRISMA flow diagram of study selection process

Initially, 4,750 articles were screened by title and abstract using Rayyan [42]. Subsequently, 142 articles were eligible for full-text screening. Backward snowball sampling from the reference lists of relevant reviews resulted in eleven additional articles being added for full-text screening. After full-text screening, nine articles met the inclusion criteria. Eight reported on depression—two on both prevalence and mean symptom scores, two on prevalence only, and four on mean scores only. Four articles reported on anxiety—two on both prevalence and mean scores, and two on prevalence only. The reported prevalence refers to the proportion of respondents scoring above a cut-off point on a self-report instrument for depression or anxiety, indicating significant depressive or anxiety symptoms and a probable disorder. Figure 1 depicts the study selection process. Table 1 provides an overview of the included articles, their characteristics, and outcome measures.

The studies examined trends in anxiety and depression among youth across seven countries. Three studies were conducted in Northern Europe (Norway, Iceland, and Finland), four in Northern America (Canada and USA), one in Asia (China) and one in Oceania (Australia). Most studies (5 out of 9) focused solely on adolescents aged 11–18 years. Four studies included young adults aged 19–25. Slightly more than (5 out of 9) employed a repeated cross-sectional design, the remaining were longitudinal. The number of pre-COVID measurements ranged from two to six. All studies included at least one measurement during the pandemic. An overview of the included studies is presented in Table 1.

### Trends in anxiety and depression symptoms before the COVID-19 pandemic

**Fig. 2:**
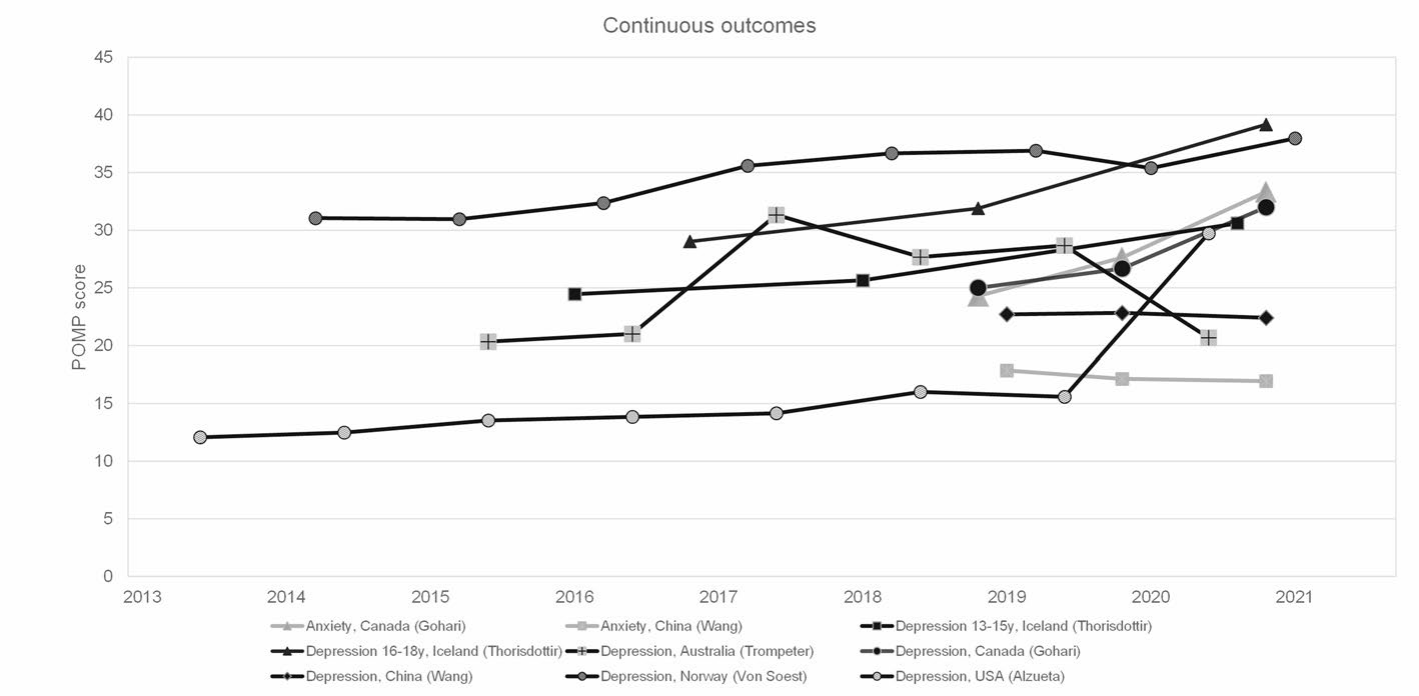
Trends in mean scores of anxiety and depressive symptoms. Mean anxiety and depressive symptom scores derived from the included studies are shown per year. Means were transformed into a comparable mean using Percentage of Maximum Possible (POMP) scores [41]. Higher POMP scores indicate more severe symptoms. Each studied population is represented by a line connecting the data points. Grey lines represent anxiety, black lines represent depression

**Fig. 3:**
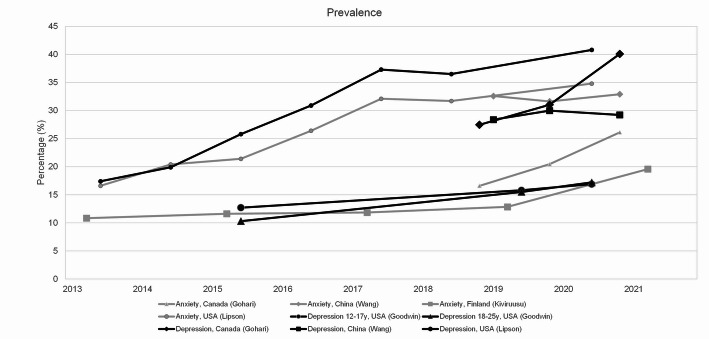
Trends in prevalence of anxiety and depression. For the various studied populations, available prevalence data are presented. Prevalence refers to the proportion of respondents scoring above a cut-off point on the respective self-report instrument for depression or anxiety, indicating (significant) depressive or anxiety symptoms and a probable depressive or anxiety disorder (see Table 1 for details). Anxiety data points are connected by grey lines; depression data points by black lines

Figures 2 and 3 illustrate the trends in depression and anxiety before and during the COVID-19 pandemic using two outcome measures: (1) mean POMP scores of anxiety and depression symptoms, and (2) prevalence, defined as the proportion of respondents scoring above the cut-off point on self-report instruments indicating significant symptoms or a probable disorder (See Table 1 for details). Most studies (7 out of 9) found increasing rates of anxiety and/or depression among youth before the pandemic. Two studies deviated from this pattern. Trompeter et al. reported a fluctuating trend, with both increases and decreases over time, and a decreasing trend in the most recent years before and during the pandemic [36]. Wang and colleagues found a stable trend in depression and anxiety before the pandemic [43].

### Trends in anxiety and depression symptoms during the COVID-19 pandemic

Trends observed during the pandemic (i.e., at the COVID-19 measurement compared to the last pre-COVID measurement) were diverse. In five out of nine studies the COVID-19 pandemic intensified pre-existing upward trends, indicating worsening of symptoms [34, 44–47]. Two studies found increases during the pandemic that were consistent with existing trends, not exceeding previous growth rates [35, 48]. Wang et al. [43] found no effect during the pandemic, aligning with the stable pre-pandemic trend. Trompeter et al. [36] observed a decrease in symptoms during the pandemic, consistent with the fluctuating trend noted earlier.

### Differences in trends between countries and populations

All studies conducted in Scandinavian countries (Norway, Iceland, and Finland) found that the pandemic amplified existing increases in depressive and anxiety symptoms among youth. North American studies (Canada and the USA) found either a continuation of increasing trends or a worsening during the pandemic. The Chinese study reported stable levels of depression and anxiety, unaffected by the pandemic. The Australian study showed an unstable trend, with a decrease during the pandemic. Several studies reported risk factors for depression and anxiety, most commonly gender (with girls/women more at risk than boys/men) and age (with either younger or older age groups within the youth population showing greater vulnerability).

## Discussion

This systematic literature review included nine studies that assessed the prevalence of depression and/or anxiety (symptoms) among adolescents and young adults (ages 12–25) both prior to and during the COVID-19 pandemic. The aim was to examine how the pandemic may have affected the long-term prevalence of depression and anxiety among youth globally.

A total of 15 study estimates were analysed—ten focusing on depression and five on anxiety—across seven countries. Except for one study from China, all were conducted in Western countries (Northern Europe, North America, and Australia).

Findings suggest that the impact of the COVID-19 pandemic on youth mental health was not uniform. Seven out of nine studies indicated that the existing trend of rising depression and anxiety levels among adolescents and young adults continued during the pandemic. Notably, five out of nine studies reported an amplified increase in anxiety and/or depression symptoms during the pandemic [34, 44–47]. The remaining studies found no pandemic-related shift in pre-existing trends, which were already increasing, stable or fluctuating [35, 36, 43, 48]. Although these findings suggest that the pandemic may have contributed to a worsening of youth mental health beyond existing trends, it remains difficult to determine the extent of this disruption. The number of suitable studies for inclusion was relatively low, limiting the ability to draw definitive conclusions.

The timing of data collection during the pandemic emerged as a key factor in explaining variability in study outcomes. Previous research has shown that mental health problems tend to increase during lockdowns and decrease when restrictions are lifted [49]– [50]. Studies that reported a negative impact of the pandemic were typically conducted during periods of stringent public health measures, including school closures, or in 2021, when it became evident that the crisis would continue with multiple waves and prolonged restrictions.

In line with this, the two studies that did not observe a continuation or amplification of rising trends collected their data during periods when public health restrictions had been eased. For example, Trompeter et al. (2022) found a decrease in mental health during the pandemic, with data collection occurring shortly after school reopenings following the first lockdown [36]. Similarly, Wang et al. [43], whose study found stable trends, collected data 5–7 months after the first wave of restrictions had been lifted.

It is also notable that the studies finding no clear effect of the pandemic on mental health trends had relatively small sample sizes compared to those reporting amplified increases.

These findings are consistent with prior research. For example, the review by Madigan et al. (2023), which compared single timepoints before and during the first year of the pandemic [27], also found amplified increases in depression and anxiety symptoms—particularly among females (consistent with our findings) and individuals from higher socioeconomic backgrounds. Regarding anxiety specifically, they reported slight increases during the pandemic . Similarly, Racine et al. [28] reported higher rates of depression and anxiety symptoms among girls and older adolescents, echoing our results. Their findings also suggested that prevalence rates continued to rise throughout the first year of the pandemic, supporting the elevated levels of depression and anxiety reported in this review for 2020 and 2021.

### Strengths and limitations

This review has several notable strengths. To the best of our knowledge, it is the first systematic literature review to examine the impact of the COVID-19 pandemic on long-term trends in depressive and anxiety symptoms among adolescents and young adults globally. A comprehensive search strategy was applied, resulting in the full-text screening of 140 articles, and no language restrictions were applied.

However, this study also has several limitations. First, all included studies relied on a single measurement during the pandemic, most of which were conducted in 2020, the first year. This may have underestimated the pandemic’s impact, as emerging evidence suggests that the most significant mental health deterioration occurred later, particularly in the final months of 2021 [27]. Further research is needed to assess whether prolonged and repeated exposure to crises increases risks for mental health problems [51, 52]. Second, most studies were conducted in Western countries, limiting the generalizability of the findings. Future research should include data from non-Western populations to better understand the global picture of youth mental health. Third, while all studies included employed random sampling, they were based on student populations, which may limit generalizability. However, given the age range targeted in this review, such samples were to be expected. Still, the main limitation of the current review is that the available data do not allow for more than a narrative analysis, relying on visual inspection. Unfortunately, we could not formally test whether factors such as age distribution, proportion of female participants, other population risk factors, or methodological characteristics of the studies—such as design, data collection duration, and analysis techniques—influenced prevalence rates, mean scores, or trends. When additional longitudinal studies with data points from before, during, and after the pandemic become available, it should be possible to conduct a more controlled meta-analysis that preferably includes more detailed information on population vulnerability factors and methodological properties.

### Implications

This review suggests that youth mental health—already in decline in many countries—may have been further negatively affected by the COVID-19 pandemic. These findings raise concerns not only for the current state of youth mental health, but also for future global crises. To monitor the magnitude and duration of these impacts, long-term surveillance of mental health is essential. We recommend prioritizing nationally representative, longitudinal studies using large, random samples. These should track mental before, during and after crises, identify at-risk groups and explore time to recovery.

This review reinforces the growing consensus that adolescents and young adults are particularly vulnerable to the psychological impacts of major crises, including pandemics. While stressors directly linked to the pandemic likely played a role, other potential contributing factors—such as cultural, socioeconomic, and institutional conditions—should be examined. Their interplay needs to be better understood to inform the development of effective prevention and intervention strategies. Despite inherent limitations, our findings highlight the urgent need to prioritize evidence-based programs and public policies focused on promoting youth mental health—not only to mitigate the effects of ongoing crises but also to build resilience for the challenges ahead.

## Conclusion

The studies included in this review—based on random samples of youth—show that symptoms of depression and anxiety were already increasing prior to the COVID-19 pandemic. While findings varied, a slight majority of the studies support the hypothesis that the pandemic exacerbated these pre-existing trends. To better understand the true impact of pandemics and other large-scale crises on youth mental health, further longitudinal studies based on random, general population samples are essential.

No animal or human studies were carried out by the authors for this article.

## Supplementary Information

Below is the link to the electronic supplementary material.

Supplementary Material 1

Supplementary Material 2

## Data Availability

All data generated or analysed during this study are included in this published article and its supplementary information files.
