# Supplementary material for: Impact of the COVID-19 pandemic on long-term trends in youth depression and anxiety
Source: Discov Ment Health. 2025 Dec 30;5(1):210. doi: 10.1007/s44192-025-00311-5 (PMC12753614; doi:10.1007/s44192-025-00311-5)
Supplement: Supplementary file 2 — Supplementary Material 2 [file 44192_2025_311_MOESM2_ESM.docx]

Tabel 1. Search terms

| Pubmed | | PsycInfo | | Embase | |
| --- | --- | --- | --- | --- | --- |
| MeSH terms | **Title & abstract**  **[tiab]** | **APA Thesaurus terms** | **Keyword search** | **Entree terms**  **/exp** | **Title/Abstract**  **:ab,ti** |
| Study Characteristics | | | | | |
| Epidemiology [Mesh:NoExp] | Epidemiolog* | Epidemiology | Epidemiolog* | epidemiology | Epidemiolog* |
| Epidemiologic Studies [Mesh:NoExp] | Incidence | Repeated Measures/ or exp posttesting/ or exp pretesting/ | Incidence | incidence | incidence |
| Incidence [Majr] | Prevalence | Cohort Analysis | Prevalence | prevalence | prevalence |
| Prevalence | Cohort stud* | exp longitudinal studies/ or exp prospective studies/ or exp follow-up studies/ or exp retrospective studies/ | Cohort stud* | longitudinal study | Longitudinal stud* |
| Cohort Studies | Cohort analysis | Time series | Temporal trend* | Prospective study | Temporal trend* |
| Interrupted Time Series Analysis | Prospective |  | Temporal variation* | follow up | Temporal variation* |
|  | Followup |  | Cohort analysis | cohort analysis | Cohort analysis |
|  | Follow-Up |  | Annual trend* | retrospective study | Annual trend* |
|  | Longitudinal |  | Annual variation* | Trend study | Annual variation* |
|  | Retrospective |  | cyclical |  | cyclical |
|  | Trend stud* |  | Prospective |  | Prospective |
|  | Time trend* |  | Followup |  | Followup |
|  | Temporal trend* |  | Follow-up |  | Follow-up |
|  | Annual trend* |  | Longitudinal |  | cohort stud* |
|  | Temporal variation* | Youth Mental Health | Retrospective |  | Retrospective |
|  | Annual variation* | Adolescent Health | Trend stud* |  | Trend stud* |
|  | cyclical | Early adolescence | Time trend* |  | Time trend* |
| Young Adult | adolescence | Late adolescence | Adolescent* | Adolescent | Adolescent* |
| Adolescent[young adult 19-24, adolescent 13-18] | Age difference* | Adolescent Psychology | adolescence | Groups by age | adolescence |
|  | Young adult* | Emerging Adulthood | Young adult* | Young adult | Young adult* |
|  | Adolescent* | Age differences | Age difference* |  | Age difference* |
|  | Depressive symptom* |  | Depressive symptom* |  | depressive symptom* |
|  | Anxiety symptom* |  | Anxiety symptom* |  | Anxiety symptom* |
|  | Anxious feeling* |  | Anxious feeling* | depression | Anxious feeling* |
| Depression | depression | Depression (Emotion) | depression | adolescent depression | depression |
| Anxiety | anxiety | Anxiety | anxiety | anxiety | anxiety |
| *Filters:*  -*Publication date: vanaf 2020* | | ***Filters:***  *- Publication date: 2020-current* | | ***Filters:***  *-Publication years: 2020, 2021, 2022, 2023, 2024* | |

**In BOLD the final search terms used**

| PubMed | |
| --- | --- |
| Epidemiology/prevalence/incidence | "Epidemiology"[MeSH Terms:noexp] OR "Epidemiologic Studies"[MeSH Terms:noexp] OR "Incidence"[MeSH Major Topic] OR "Prevalence"[MeSH Terms] OR "epidemiolog*"[Title/Abstract] OR "Incidence"[Title/Abstract] OR "Prevalence"[Title/Abstract]  2,131,691 |
| Meerdere meetmomenten | "Cohort Studies"[MeSH Terms] OR "Interrupted Time Series Analysis"[MeSH Terms] OR "cohort stud*"[Title/Abstract] OR "cohort analysis*"[Title/Abstract] OR "prospective"[Title/Abstract] OR "followup"[Title/Abstract] OR "follow up"[Title/Abstract] OR "longitudinal"[Title/Abstract] OR "retrospective"[Title/Abstract] OR "trend stud*"[Title/Abstract] OR "time trend*"[Title/Abstract] OR "temporal trend*"[Title/Abstract] OR "temporal variation*"[Title/Abstract] OR "annual trend*"[Title/Abstract] OR "annual variation*"[Title/Abstract] OR "cyclical"[Title/Abstract]  3,808,550 |
| Jeugd/jongvolwassenen | "Young Adult"[MeSH Terms] OR "Adolescent"[MeSH Terms] OR "young adult*"[Title/Abstract] OR "adolescent*"[Title/Abstract] OR "age difference*"[Title/Abstract] OR "adolescence*"[Title/Abstract]  2,849,988 |
| Depressieve/angst gevoelens | "depression"[MeSH Terms] OR "anxiety"[MeSH Terms] OR "depressive symptom*"[Title/Abstract] OR "anxiety symptom*"[Title/Abstract] OR "anxious feeling*"[Title/Abstract] OR "depression"[Title/Abstract] OR "anxiety*"[Title/Abstract]  652,558 |
| **Combined to one search term (without NOT) + filter for date of publication** | **(("Epidemiology"[MeSH Terms:noexp] OR "Epidemiologic Studies"[MeSH Terms:noexp] OR "Incidence"[MeSH Major Topic] OR "Prevalence"[MeSH Terms] OR "epidemiolog*"[Title/Abstract] OR "Incidence"[Title/Abstract] OR "Prevalence"[Title/Abstract]) AND ("Cohort Studies"[MeSH Terms] OR "Interrupted Time Series Analysis"[MeSH Terms] OR "cohort stud*"[Title/Abstract] OR "cohort analysis*"[Title/Abstract] OR "prospective"[Title/Abstract] OR "followup"[Title/Abstract] OR "follow up"[Title/Abstract] OR "longitudinal"[Title/Abstract] OR "retrospective"[Title/Abstract] OR "trend stud*"[Title/Abstract] OR "time trend*"[Title/Abstract] OR "temporal trend*"[Title/Abstract] OR "temporal variation*"[Title/Abstract] OR "annual trend*"[Title/Abstract] OR "annual variation*"[Title/Abstract] OR "cyclical"[Title/Abstract]) AND ("Young Adult"[MeSH Terms] OR "Adolescent"[MeSH Terms] OR "young adult*"[Title/Abstract] OR "adolescent*"[Title/Abstract] OR "age difference*"[Title/Abstract] OR "adolescence*"[Title/Abstract]) AND ("depression"[MeSH Terms] OR "anxiety"[MeSH Terms] OR "depressive symptom*"[Title/Abstract] OR "anxiety symptom*"[Title/Abstract] OR "anxious feeling*"[Title/Abstract] OR "depression"[Title/Abstract] OR "anxiety*"[Title/Abstract])) AND (2020:2024[pdat])**  **1,510** |

| PsychInfo | |
| --- | --- |
| Epidemiology/prevalence/incidence | 1  epidemiology/  56772    2  epidemiolog*.mp. [mp=title, abstract, heading word, table of contents, key concepts, original title, tests & measures, mesh word]  121299    3  incidence.mp. [mp=title, abstract, heading word, table of contents, key concepts, original title, tests & measures, mesh word]  66135    4  prevalence.mp. [mp=title, abstract, heading word, table of contents, key concepts, original title, tests & measures, mesh word]  154261    5  1 or 2 or 3 or 4  282864 |
| Meerdere meetmomenten | 6  repeated measures/ or posttesting/ or pretesting/  1696    7  cohort analysis/  1712    8  exp longitudinal studies/ or exp prospective studies/ or exp followup studies/ or exp retrospective studies/  30632    9  time series/  2714    10  cohort stud*.mp. [mp=title, abstract, heading word, table of contents, key concepts, original title, tests & measures, mesh word]  50814    11  prospective.mp. [mp=title, abstract, heading word, table of contents, key concepts, original title, tests & measures, mesh word]  87375    12  followup.mp. [mp=title, abstract, heading word, table of contents, key concepts, original title, tests & measures, mesh word]  19462    13  follow-up.mp. [mp=title, abstract, heading word, table of contents, key concepts, original title, tests & measures, mesh word]  168630    14  longitudinal.mp. [mp=title, abstract, heading word, table of contents, key concepts, original title, tests & measures, mesh word]  169728    15  retrospective.mp. [mp=title, abstract, heading word, table of contents, key concepts, original title, tests & measures, mesh word]  62406    16  trend stud*.mp. [mp=title, abstract, heading word, table of contents, key concepts, original title, tests & measures, mesh word]  166    17  time trend*.mp. [mp=title, abstract, heading word, table of contents, key concepts, original title, tests & measures, mesh word]  1558    18  temporal trend*.mp. [mp=title, abstract, heading word, table of contents, key concepts, original title, tests & measures, mesh word]  839  19  temporal variation*.mp. [mp=title, abstract, heading word, table of contents, key concepts, original title, tests & measures, mesh word]  863  20  cohort analysis.mp. [mp=title, abstract, heading word, table of contents, key concepts, original title, tests & measures, mesh word]  2550  21  annual trend*.mp. [mp=title, abstract, heading word, table of contents, key concepts, original title, tests & measures, mesh word]  65  22  annual variation*.mp. [mp=title, abstract, heading word, table of contents, key concepts, original title, tests & measures, mesh word]  114  23  cyclical.mp. [mp=title, abstract, heading word, table of contents, key concepts, original title, tests & measures, mesh word]  3078  24  6 or 7 or 8 or 9 or 10 or 11 or 12 or 13 or 14 or 15 or 16 or 17 or 18 or 19 or 20 or 21 or 22 or 23  448447 |
| jeugd/jongvolwassenen | 25  youth mental health/  2339    26  adolescent health/  4019    27  late adolescence/  478    28  adolescent psychology/  5843    29  emerging adulthood/  8238    30  age differences/  85685    31  "adolescent*".mp. [mp=title, abstract, heading word, table of contents, key concepts, original title, tests & measures, mesh word]  499354    32  "adolescence".mp. [mp=title, abstract, heading word, table of contents, key concepts, original title, tests & measures, mesh word]  87961    33  "young adult*".mp. [mp=title, abstract, heading word, table of contents, key concepts, original title, tests & measures, mesh word]  217237    34  "age difference*".mp. [mp=title, abstract, heading word, table of contents, key concepts, original title, tests & measures, mesh word]  89898    35  25 or 26 or 27 or 28 or 29 or 30 or 31 or 32 or 33 or 34  703328 |
| Depressieve/angst gevoelens | 36  "depression (emotion)"/  27117    37  anxiety/  79530    38  depressive symptom*.mp. [mp=title, abstract, heading word, table of contents, key concepts, original title, tests & measures, mesh word]  64508    39  anxiety symptom*.mp. [mp=title, abstract, heading word, table of contents, key concepts, original title, tests & measures, mesh word]  15676    40  anxious feeling*.mp. [mp=title, abstract, heading word, table of contents, key concepts, original title, tests & measures, mesh word]  140    41  depression.mp. [mp=title, abstract, heading word, table of contents, key concepts, original title, tests & measures, mesh word]  395729    42  anxiety.mp. [mp=title, abstract, heading word, table of contents, key concepts, original title, tests & measures, mesh word]  294409    43  36 or 37 or 38 or 39 or 40 or 41 or 42  559218 |
| **Combined to one search term (without NOT) + filter for date of publication** | **54**  **5 and 24 and 35 and 43**  **7578**    **55**  **limit 54 to yr="2020 -Current"**  **1158**  **>>>**  **(epidemiology/ or epidemiolog*.mp. or incidence.mp. or prevalence.mp.) and (repeated measures/ or posttesting/ or pretesting/ or cohort analysis/ or exp longitudinal studies/ or exp prospective studies/ or exp followup studies/ or exp retrospective studies/ or time series/ or cohort stud*.mp. or prospective.mp. or followup.mp. or follow-up.mp. or longitudinal.mp. or retrospective.mp. or trend stud*.mp. or time trend*.mp. or temporal trend*.mp. or temporal variation*.mp. or cohort analysis.mp. or annual trend*.mp. or annual variation*.mp. or cyclical.mp.) and (youth mental health/ or adolescent health/ or late adolescence/ or adolescent psychology/ or emerging adulthood/ or age differences/ or "adolescent*".mp. or "adolescence".mp. or "young adult*".mp. or "age difference*".mp.) and ("depression (emotion)"/ or anxiety/ or depressive symptom*.mp. or anxiety symptom*.mp. or anxious feeling*.mp. or depression.mp. or anxiety.mp.)**  **limit 1 to yr="2020 -Current"** |

| Embase | |
| --- | --- |
| Epidemiology/prevalence/incidence | #7  #1 OR #2 OR #3 OR #4 OR #5 OR #6  3,316,913  #6  'prevalence':ab,ti  1,165,600  #5  'incidence':ab,ti  1,372,332  #4  'epidemiolog*':ab,ti  596,316  #3  'prevalence'/de  972,944  #2  'incidence'/de  591,488  #1  'epidemiology'/de  263,871 |
| Meerdere meetmomenten | #8 OR #9 OR #10 OR #11 OR #12 OR #13 OR #14 OR #15 OR #16 OR #17 OR #18 OR #19 OR #20 OR #21 OR #22 OR #23 OR #24 OR #25 OR #26 OR #27  5,761,742  #27  'cyclical':ab,ti  11,758  #26  'annual variation*':ab,ti  1,933  #25  'annual trend*':ab,ti  1,399  #24  'cohort analysis':ab,ti  18,477  #23  'temporal variation*':ab,ti  11,816  #22  'temporal trend*':ab,ti  18,101  #21  'time trend*':ab,ti  14,660  #20  'trend stud*':ab,ti  759  #19  'retrospective':ab,ti  1,300,147  #18  'cohort stud*':ab,ti  486,010  #17  'follow-up':ab,ti  1,986,629  #16  'followup':ab,ti  1,777,926  #15  'prospective':ab,ti  1,129,373  #14  'longitudinal':ab,ti  455,872  #13  'trend study'/de  62,834  #12  'retrospective study'/de  1,552,424  #11  'cohort analysis'/de  1,102,667  #10  'follow up'/de  2,143,768  #9  'prospective study'/de  901,081  #8  'longitudinal study'/de  204,393 |
| Jeugd jongvolwassenen | #36  #29 OR #30 OR #31 OR #32 OR #33 OR #34 OR #35  2,587,070  #35  'age difference*':ab,ti  13,758  #34  'young adult*':ab,ti  165,244  #33  'adolescence':ab,ti  91,505  #32  'adolescent*':ab,ti  432,667  #31  'young adult'/de  528,025  #30  'groups by age'/de  129,954  #29  'adolescent'/de  1,956,885 |
| Depressieve/angst gevoelens | ZONDER NOT  #45  #37 OR #38 OR #39 OR #40 OR #41 OR #42 OR #43 OR #44  1,032,113  #44  'anxiety':ab,ti  382,105  #43  'depression':ab,ti  592,512  #42  'anxious feeling*':ab,ti  111  #41  'anxiety symptom*':ab,ti  20,945  #40  'depressive symptom*':ab,ti  94,202  #39  'anxiety'/de  300,744  #38  'adolescent depression'/de  1,861  #37  'depression'/de  509,028 |
| **Combined to one search term (without NOT) + filter for date of publication** | **#59**  **#58 AND (2020:py OR 2021:py OR 2022:py OR 2023:py OR 2024:py)**  **3,532**  **#58**  **#7 AND #28 AND #36 AND #45**  **9,132**  **>>>**  **('epidemiology'/de OR 'incidence'/de OR 'prevalence'/de OR 'epidemiolog*':ab,ti OR 'incidence':ab,ti OR 'prevalence':ab,ti) AND ('longitudinal study'/de OR 'prospective study'/de OR 'follow up'/de OR 'cohort analysis'/de OR 'retrospective study'/de OR 'trend study'/de OR 'longitudinal':ab,ti OR 'prospective':ab,ti OR 'followup':ab,ti OR 'follow-up':ab,ti OR 'cohort stud*':ab,ti OR 'retrospective':ab,ti OR 'trend stud*':ab,ti OR 'time trend*':ab,ti OR 'temporal trend*':ab,ti OR 'temporal variation*':ab,ti OR 'cohort analysis':ab,ti OR 'annual trend*':ab,ti OR 'annual variation*':ab,ti OR 'cyclical':ab,ti) AND ('adolescent'/de OR 'groups by age'/de OR 'young adult'/de OR 'adolescent*':ab,ti OR 'adolescence':ab,ti OR 'young adult*':ab,ti OR 'age difference*':ab,ti) AND ('depression'/de OR 'adolescent depression'/de OR 'anxiety'/de OR 'depressive symptom*':ab,ti OR 'anxiety symptom*':ab,ti OR 'anxious feeling*':ab,ti OR 'depression':ab,ti OR 'anxiety':ab,ti) AND [2020-2024]/py** |
